# Pyrexia in a Patient with Megaloblastic Anemia: A Case Report and Literature Review

**Published:** 2013-06

**Authors:** Kevin Manuel, Somanath Padhi, Renu G’Boy Varghese

**Affiliations:** Department of Pathology, Pondicherry Institute of Medical Sciences, Ganapathychettykulam, Puducherry, India

**Keywords:** Megaloblastic anemia, Pyrexia, Vitamin B12, Folic acid

## Abstract

Deficiency of vitamin B_12 _and/or folic acid as a cause of pyrexia, though known, is rarely reported in literature. We aimed to report a case in a 51 year old woman, who presented with fever and pancytopenia and was diagnosed to have megaloblastic anemia secondary to vitamin B_12_ and folate deficiency. The pyrexia subsided following the intramuscular injection of vitamin B_12_ and oral folic acid administration. All the other infective, inflammatory/autoimmune, endocrine causes of pyrexia were excluded by appropriate investigations. Therefore, we suggest that all physicians be aware of megaloblastic anemia as a treatable cause of pyrexia in order to avoid unnecessary costly investigations and antibiotic usage.

## Introduction

Megaloblastic anemias are a group of disorders characterized by peripheral blood cytopenia(s) resulting due to ineffective hematopoiesis in the marrow. They are usually caused by nutritional deficiencies (most common) of either vitamin B_12 _or folate; or both, inherited disorders of DNA synthesis, or following certain drug therapy.^1^ Pyrexia in megaloblastic anemia, albeit well known, is rarely characterized. However, megaloblastic anemia, solely as the cause of pyrexia, can be found in only a small proportion of cases, for which differentiation from fever of unknown origin (FUO) may be difficult even after exhaustive laboratory investigations.^2^^-^^8^

The aim of the present article was to highlight this aspect of megaloblastic anemia with a brief review of the existing literature and create awareness among practicing physicians about a treatable condition.

## Case Presentation

A 51 year old lady, vegetarian, presented to the General Medicine Outpatient Department of Pondicherry Institute of Medical Sciences, Puducherry, India, with complaints of fever, nausea with vomiting, and burning micturition of 3 days’ duration. The fever was on and off, moderate grade, and not associated with chills and rigors. She had easy fatigability with loss of weight and appetite. The patient had a history of jaundice 3 months previously, for which she had taken ayurvedic medications and her symptoms had resolved within a month. The patient had attained menopause 3 months earlier, before which she had regular cycles. There was no history of cough with expectoration, headache, arthralgia, and rash, and nor was there a history of recent travel to malaria endemic zone or exposure to any patient of tuberculosis.

Examination of the patient revealed pulse rate of 118 per minute, blood pressure of 110/70 mm Hg (supine), and oral temperature of 38.3°C (101°F). She had moderate conjunctival pallor and scleral icterus. There was no lymphadenopathy, clubbing, eschar, or skin rashes. Oxygen saturation was 99% on room air, and there were no signs of respiratory distress in the patient. Cardiovascular examination revealed a systolic flow murmur in the aortic area. The respiratory and nervous system examinations were within normal limits. Her chest X-ray and abdomen ultrasound revealed no significant abnormalities except for mild hepatomegaly.

**Figure 1:**
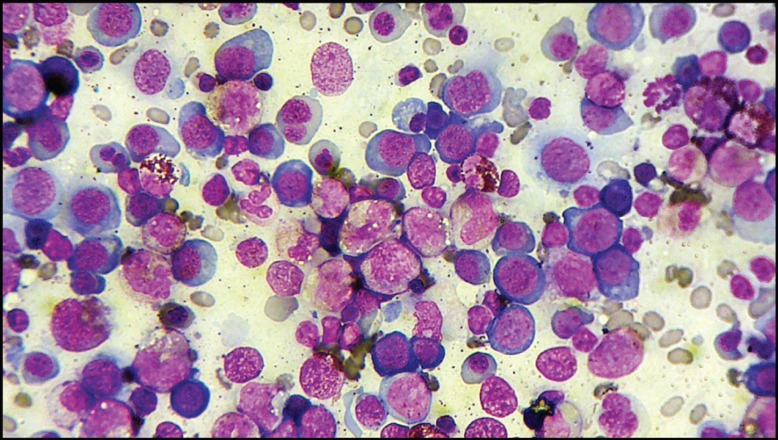
Bone marrow aspirate smear shows markedly increased cellularity with erythroid hyperplasia and trilineage dyspoiesis (Wright-Giemsa, ×400

**Figure 2:**
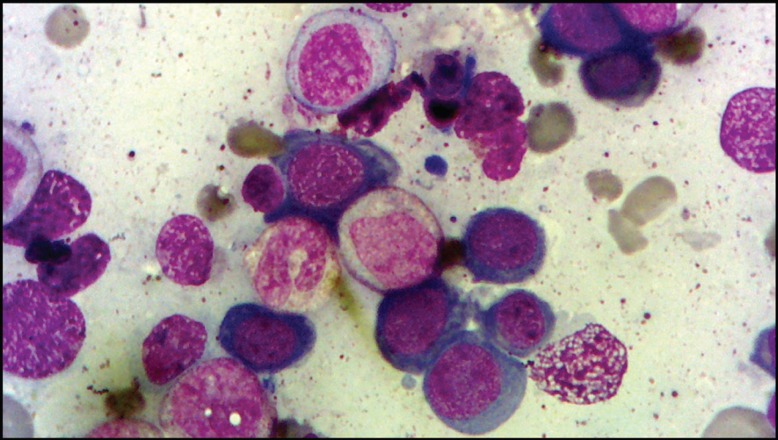
Bone marrow aspirate smear demonstrates numerous megaloblasts and giant metamyelocytes (Wright-Giemsa, ×1000).

Routine hematological evaluation, on admission, revealed very low hemoglobin (Hb); 22 g/L (120-160), hematocrit; 7.2% (35-45), total leukocyte count (TLC); 3×10^9^/L (4-11), total platelet count (TPC); 64.5×10^9^/L (150-450), absolute neutrophil count; 1.9×10^9^/L (1.5-8×10^9^/L), corrected reticulocyte count; 1.5% (0.5-2), red cell distribution width; 17.5% (11.5-14.5), mean corpuscular volume (MCV); 114.3 fL (80-98), mean corpuscular hemoglobin (MCH); 34.9 pg (26-32), and mean corpuscular hemoglobin concentration (MCHC); 30.6% (32-36). Peripheral smear showed pancytopenia with a moderate degree of anisopoikilocytosis and a good number of macrocytes, macro-ovalocytes, and hypersegmented neutrophils. Bone marrow aspiration and trephine biopsy from the right posterior superior iliac spine revealed marked hypercellularity for age (70%), florid erythroid hyperplasia with an altered myeloid to erythroid ratio (1:2), megaloblastic dyspoiesis, and numerous giant metamyelocytes. Micromegakaryocytes and/or megakaryocyte clustering were not seen. Perl stain showed adequate marrow iron stores without any ring sideroblasts. There was no evidence of blast prominence (4%), granulomas, hemoparasites, malignancy, or increased reticulin condensation (figures 1 and 2). The bone marrow morphology was suggestive of megaloblastic anemia, which was confirmed biochemically by low levels of serum vitamin B_12_ (59.6 pg/mL, reference; 180- 900), low normal folic acid (3.9 ng/mL, reference; 4-24), and markedly elevated serum lactate dehydrogenase (LDH) [10,550 IU/L, reference; 225-420]. The patient’s routine liver and renal function tests were within normal limits except for mild unconjugated hyperbilirubinemia (total bilirubin; 4.8 mg/dL [0.2-1.2]/direct; 0.4 mg/dL [up to 0.3]). Her upper and lower gastrointestinal endoscopy did not show any abnormality.

Her routine microbiological (aerobic and anaerobic culture), serological, autoimmune, inflammatory (serum C-reactive protein; 3mg/L, ref.<10mg/L), and endocrine work-up were negative. Normal viral titers along with the absence of reactive lymphocytes in the peripheral smear ruled out the possible viral etiology.

**Clinical Progression**

**Figure 3:**
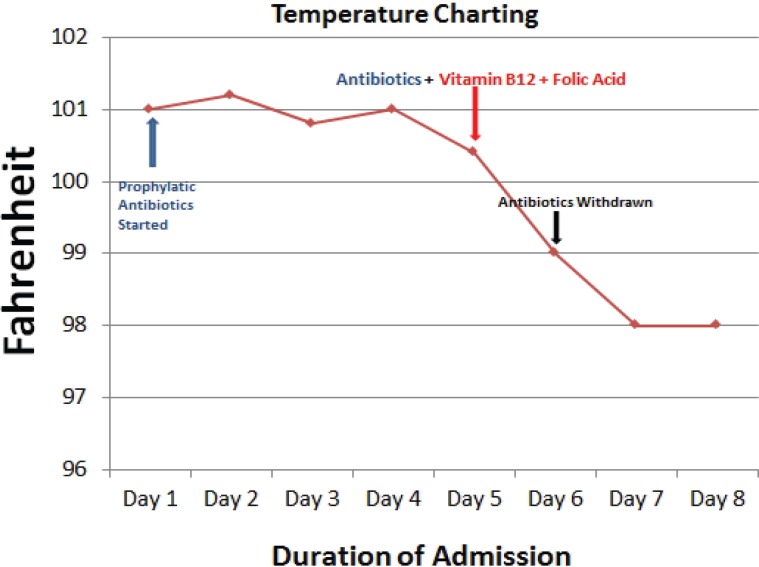
Line Chart shows the patient’s temperature during the course of illness in the hospital.

Pending laboratory investigation reports, and in view of neutropenia, the patient was started prophylactically on broad spectrum intravenous antibiotics (on day 2) based on the protocol of infection management for a period of 4 days but the patient continued to be pyrexial. Therefore, in view of the positive laboratory investigations pointing towards megaloblastic anemia along with the absence of any positive microbiological findings, the patient was started on injection vitamin B_12_ and oral folic acid (on day 5) along with on-going parenteral antibiotics. Pyrexia settled on day 6 of admission with just vitamin B_12_ and folic acid therapy and, consequently, the antibiotics were withdrawn (figure 3).

Given the patient’s low hemoglobin, she was transfused with 3 units of packed cell volume. The patient improved symptomatically after being prescribed vitamin B_12_ and folic acid supplements, following which the patient was discharged in a stable condition. Routine follow-up (at one month) showed normalization of vitamin B_12_ (656 pg/mL) and folate (>5 ng/mL) levels as well as improvement in hematological parameters (hemoglobin; 80 g/L, MCV; 86fL) without any febrile episodes.

## Discussion

Our patient’s dramatic response to nutritional supplements in our case supports the notion that the pyrexia was attributable directly to megaloblastic anemia secondary to vitamin B_12_ and folate deficiency rather than anything else, as was ruled out by appropriate available diagnostic modalities. As per the modified Petersdorf criteria,^2^ FUO is defined as: 1) a temperature exceeding 38.3°C; 2) duration of the fever of more than three weeks; and 3) evaluation of three outpatient visits or three days in hospital. Our patient satisfied two out of the three criteria (1 and 3).

In a study by Tahlan et al.^3^ the incidence of low-grade fever in nutritional megaloblastic anemia varied from 28% to 60% (259 of 509 patients). Another study from Northern India described persistent low-grade fever in 70% of the females with B_12 _and/or folate deficiency.^4^ McKee,^5^ reviewed 122 patients of nutritional megaloblastic anemia for the presence of pyrexia (temperature≥37.8°C [100°F]). In 49/122 (40%), pyrexia was attributable solely to the megaloblastic disease. In addition, the majority of the patients had a minimal rise of temperature (≤38.5°C). Only occasionally the values were above 38.5°C (102°F), and rarely were they greater than 40°C (4/49, 8%). Those with severe disease characterized by high MCV, low hematocrit (<20%), low thrombocytopenia (<100×10^9 ^/L), high LDH (>1000 IU/L), and unconjugated hyperbilirubinemia (>1.5 mg/dL) were more likely to be febrile. In the majority of the patients, fever subsided 24 to 72 hours after supplementation of vitamin B_12_ and/or folate, suggesting the rapid correction of ineffective hematopoiesis.

A comparative review of literature highlighting pyrexia in megaloblastic anemia is presented in table 1. Carpenter et al.^6^ described a 38 year old female patient who presented with chronic, low-grade intermittent fever (37.8°C), mild macrocytosis (MCV=104 fL), and normal hematocrit secondary to chronic atrophic gastritis, low vitamin B_12_ (115 pg/mL, reference range: 190-900 pg/mL), and co-existent proximal intestinal type gastric adenocarcinoma. The pathophysiological mechanism for her pyrexia could have been attributed to either nutritional deficiency secondary to chronic atrophic gastritis of pernicious anemia or release of tumoral cytokines (Interleukin-6); or both. However, response to vitamin B_12_ supplementation therapy was not documented in that case, and the patient expired due to metastatic disease following gastrectomy. Negi et al.^7^ reported a case of anicteric male with pyrexia (39.7°C), bicytopenia, and macrocytosis (MCV=105 fL) secondary to B_12_ deficiency (105 pg/mL). Singanayagam et al.^8^ reported a young male with pyrexia of 6 weeks’ duration (38.8°C), severe pancytopenia, and mild hyperbilirubinemia secondary to folate deficiency (1.2 ng/mL, reference range: 5-24 ng/mL) and low normal vitamin B_12_ (202 pg/mL). The present report described a case of megaloblastic anemia in a middle-aged female patient, who presented with low-grade pyrexia, pancytopenia, macrocytosis (114.3 fL), very high LDH (10,550 IU/L, reference range: 225-420 IU/L), and mild unconjugated hyperbilirubinemia; secondary to combined deficiency of B_12_ (59.6 pg/mL) and folate (3.9 ng/mL). In all the three cases (including the present one) as was described above, pyrexia subsided 24 to 72 hours after initiation of supplementation therapy.

**Table 1:** Comparison of the present case of pyrexia and megaloblastic anemia with similar cases published in the literature

**Author, year, (no. of cases)**	**Age, sex**	**Fever**		**Hemogram**				**Pathology**	**Response to vitamin supplement **
		**Duration**	**Temp.**	**Peripheral blood smear**	**MCV (80-98fL)**	**Serum B**_12_ ** (190-900 pg/mL)**	**Serum folate (5-24 ng/mL)**		
Carpenter et al.^6^ 2000, (1)	38, Female	6 ½ weeks, persistent, low grade, dominant presentation	37.8°C	Macrocytosis (Hct; 42.7%, TLC; 9.1×10^9^/L, TPC; 2.7×10^9^/L)	104	115	Normal	Pernicious anemia, gastric adenocarcinoma (GE^#^ junction), bone marrow not done	Not assessed, death 4 months post gastrectomy
Negi et al.^7^ 2011, (1)	18, Male	3 days	103.6°F (39.7°C)	Bicytopenia (Hb; 38g/L, TLC; 3.2×10^9^/L, TPC; WNL)	105	105	5.05	Dimorphic bone marrow picture, mild splenomegaly, no icterus	Afebrile after 72 hours
Singanayagam et al.^8^ 2008, (1)	29, Male	6 weeks	38.8°C	Pancytopenia (Hb; 28g/L, TLC; 2.6×10^9^/L, TPC; 26 ×10^9^/L)	112	202 (low normal)	1.2	Megaloblastic anemia (bone marrow), splenomegaly, mild unconjugated hyperbilirubinemia	Afebrile after 48 hours (B_12 _and Folate)
Present case, 2012, (1)	51, Female	1 week	38°C	Pancytopenia (Hb;22g/L,TLC; 3×10^9^/L, TPC; 64.5×10^9^/L)	114.3	59.6	3.9	Megaloblastic anemia (bone marrow), mild hepatomegaly, unconjugated hyperbilirubinemia, high lactate dehydrogenase	Afebrile after 24 hours (B_12_ and Folate)

MCV: mean corpuscular volume; Hct: hematocrit; TLC: total leukocyte count; TPC: total platelet count; #: gastro-esophageal junction; Hb: hemoglobin; WNL: within normal limit

The exact cause of fever in megaloblastic anemia is unknown and at present, seems more hypothetical rather than conclusive. Association of pyrexia and megaloblastic anemia appears to be causal, whereas in other types of anemias, it seems more coincidental. Megaloblastic anemia is a panmyelosis, characterized by hypercellular marrow and ineffective hematopoiesis. Premature destruction of hematopoietic precursors possibly releases intracellular substances, which might function as systemic pyrogens. As was suggested by the researchers, dramatic response to B_12_ and/or folate supplementation (within 24 to 72 hours) strongly supports the above-said hypothesis. Alternatively, the defective oxygenation at the thermoregulatory center of the hypothalamus might be the explanation for pyrexia. However, lack of correlation between neurological manifestation and pyrexia in megaloblastic disease does not support this theory.^5^ Moreover, studies have also shown that a rise in temperature might cause depletion of folate stores, both in red blood cells and serum, leading to disturbance of folate metabolism. So whether pyrexia is the cause of folate deficiency or vice versa is yet to be fully understood.^9^

## Conclusion

All patients presenting with pyrexia and cytopenia should be carefully evaluated for possible vitamin B_12_ and folate deficiency in order to prevent the unnecessary use of antibiotics. Larger studies highlighting the possible role of cytokine signaling and bone marrow stromal microenvironment might throw some light in understanding the pathophysiological mechanism of pyrexia in megaloblastic anemia.
